# Performance of a real-time PCR approach for diagnosing *Schistosoma haematobium* infections of different intensity in urine samples from Zanzibar

**DOI:** 10.1186/s40249-020-00726-y

**Published:** 2020-09-04

**Authors:** Dominique Keller, Julian Rothen, Jean-Pierre Dangy, Corina Saner, Claudia Daubenberger, Fiona Allan, Shaali M. Ame, Said M. Ali, Fatma Kabole, Jan Hattendorf, David Rollinson, Ralf Seyfarth, Stefanie Knopp

**Affiliations:** 1 Biolytix AG, Benkenstrasse 254, 4108 Witterswil, Switzerland; 2 Swiss Tropical and Public Health Institute, Socinstrasse 57, 4002 Basel, Switzerland; 3 University of Basel, Petersplatz 1, 4001 Basel, Switzerland; 4 Wolfson Wellcome Biomedical Laboratories, Department of Life Sciences, Natural History Museum, Cromwell Road, London, SW7 5BD UK; 5 Public Health Laboratory Ivo de Carneri, P.O. Box 122, Chake-Chake, Pemba United Republic of Tanzania; 6 Neglected Diseases Programme, Ministry of Health, P.O. Box 236, Zanzibar Town, Unguja United Republic of Tanzania

**Keywords:** Control, Diagnosis, *Dra 1*, Elimination, Microhaematuria, Real-time PCR, *Schistosoma haematobium*, Surveillance, Urine filtration, Zanzibar

## Abstract

**Background:**

Efforts to control and eliminate schistosomiasis have accelerated over the past decade. As parasite burden, associated morbidity and egg excretion decrease, diagnosis with standard parasitological methods becomes harder. We assessed the robustness and performance of a real-time PCR (qPCR) approach in comparison with urine filtration microscopy and reagent strip testing for the diagnosis of *Schistosoma haematobium* infections of different intensities.

**Methods:**

The robustness of DNA isolation and qPCR was validated in eight laboratories from Europe and Africa. Subsequently, 792 urine samples collected during cross-sectional surveys of the Zanzibar Elimination of Schistosomiasis Transmission (ZEST) project in 2012–2017 were examined with qPCR in 2018. Diagnostic sensitivity of the qPCR was calculated at different infection intensity categories, using urine filtration microscopy as reference test. Spearman’s rank correlation between Ct-values and *S. haematobium* egg counts was assessed and Ct-value percentiles for infection intensity categories determined.

**Results:**

*S. haematobium Dra1* DNA-positive samples were identified correctly in all eight laboratories. Examination of urine samples from Zanzibar revealed *Dra1* DNA in 26.8% (212/792) by qPCR, *S. haematobium* eggs in 13.3% (105/792) by urine filtration, and microhaematuria in 13.8% (109/792) by reagent strips. Sensitivity of the qPCR increased with augmenting egg counts: 80.6% (29/36) for counts between 1 and 4 eggs, 83.3% (15/18) for counts between 5 and 9 eggs, 100% (23/23) for counts between 10 and 49 eggs, and 96.4% (27/28) for counts of 50+ eggs. There was a significant negative correlation between Ct-values and egg counts (Spearman’s rho = − 0.49, *P* < 0.001). Seventy-five percent of the Ct-values were ≥ 33 in the egg-negative category, < 31 in the light intensity category, and < 24 in the heavy intensity category.

**Conclusions:**

While the sensitivity of the qPCR was ~ 80% for very light intensity infections (egg counts < 10), in general, the *Dra1* based qPCR assay detected twice as many *S. haematobium* infections compared with classical parasitological tests. The qPCR is hence a sensitive, urine-based approach for *S. haematobium* diagnosis that can be used for impact assessment of schistosomiasis elimination programmes, individual diagnosis, and in improved format also for verification and certification of elimination.

**Trial registration:**

ISRCTN, ISRCTN48837681. Registered 05 September 2012 - Retrospectively registered.

## Background

Schistosomiasis is a debilitating disease that particularly affects poor and economically deprived populations in 78 endemic countries worldwide [1]. In 2017, the global burden due to schistosomiasis was estimated at 1.43 million disability adjusted life years [2]. The World Health Organization (WHO) envisions a world free of schistosomiasis and urged their member states to scale up interventions with the goal to control the morbidity caused by infections with the blood fluke of the genus *Schistosoma* by 2020, to achieve elimination as a public health problem by 2025, and to interrupt transmission in selected countries by 2025 [1, 3, 4]. Over the past decade, efforts of schistosomiasis control programmes and drug donations accelerated, the number of people treated for schistosomiasis increased considerably, and in 2017, the coverage of school-aged children living in endemic countries targeted with praziquantel reached 68% [5].

There are several countries considered endemic for schistosomiasis that have successfully controlled morbidity, eliminated the disease as a public health problem, and even have interrupted transmission [6]. Most of these countries regularly treated infected individuals or the whole at-risk population for schistosomiasis, applied additional measures such as intermediate host snail control, environmental modification, health education and behaviour change interventions, and often experienced improvements of the socio-economic standard over time [6]. Moving from morbidity control towards elimination as public health problem, and finally interruption of transmission in endemic countries goes along with reductions in the prevalence and intensity of infection. As worm burdens decrease, fewer eggs are produced and excreted in urine (*S. haematobium*) or stool (other *Schistosoma* species). Lighter and asymptomatic infections are more difficult to diagnose and classical parasitological tests such as the reagent strip method to detect blood in urine as a proxy for *S. haematobium* infection or microscopy to detect *Schistosoma* eggs in urine or stool start to miss infections [7, 8]. More sensitive diagnostic tests are required to monitor the impact of elimination efforts in areas where prevalence and infection intensity are low, for surveillance of potentially recrudescing transmission, and for verification of interruption of transmission [9–11].

Zanzibar is among the first settings in sub-Saharan Africa where elimination of urogenital schistosomiasis seems feasible and where elimination efforts implemented since 2012 have successfully reduced the prevalence and infection intensity to very low levels [12, 13].

Here, we assessed the robustness and performance of a standardized real-time polymerase chain reaction (qPCR) approach for the detection of the *S. haematobium* specific *Dra1* DNA repeat in urine samples from Zanzibar. First, the robustness of DNA isolation from urine samples and the qPCR assay system were verified in a ring trial including a total of eight hospital, industry, and academic research laboratories from Europe and Africa. Subsequently, a selection of 792 urine samples from participants of the Zanzibar Elimination of Schistosomiasis Transmission (ZEST) project [14] that had been examined previously with reagent strips and urine filtration microscopy in Zanzibar between 2011 and 2017, were analysed by qPCR in Switzerland in 2018 and the performance of the qPCR system was evaluated. These selected samples represented a spectrum of egg counts to allow evaluation of the assay in relationship to intensity of infection.

## Methods

### Study area

Unguja and Pemba islands are the largest islands comprised in the Zanzibar archipelago offshore mainland Tanzania. The islands’ projected population for 2019 was 1.6 million inhabitants [15].

Urogenital schistosomiasis caused by *S. haematobium* was highly endemic in the last century, with more than half of the school-aged population being infected in high-risk areas [16–18]. Intense morbidity control efforts with regular mass drug administration (MDA) campaigns implemented since the early 2000s and elimination interventions that started in 2011 with the ZEST project [14, 19–21], reduced the overall *S. haematobium* prevalence to < 2% and heavy infection intensities to < 1% in schoolchildren and adults in 2017 [12, 13].

### The ZEST project

Efforts to eliminate urogenital schistosomiasis from Zanzibar started in 2011 with the formation of the ZEST alliance and strong political commitment from the Zanzibar government. The ZEST alliance included the Neglected Diseases Programme of the Zanzibar Ministry of Health (MoH), the Public Health Laboratory-Ivo de Carneri (PHL-IdC), the WHO, the Schistosomiasis Control Initiative, the Bill & Melinda Gates Foundation, the Schistosomiasis Consortium for Operational Research and Evaluation (SCORE), the Natural History Museum in London, the Swiss Tropical and Public Health Institute and other partners and stakeholders [14]. The Zanzibar MoH started biannual MDA of praziquantel in almost all communities and schools in Unguja and Pemba in 2012. In a cluster randomized trial funded by SCORE and implemented from 2011 to 2017, the impact of biannual MDA alone and in combination with snail control or behaviour change interventions was studied and compared [12–14]. One of the secondary objectives of the trial was to contribute to the validation and to assess the performance of new techniques for the diagnosis of *S. haematobium*.

### Field procedures

Annual cross-sectional parasitological surveys were conducted in 91 study schools and 91 study communities, respectively, in Zanzibar (Pemba and Unguja islands), from November 2011 to May 2017. Every year and in each study location, urine samples were collected from ~100 randomly selected students aged 9–12 years and from ~50 randomly selected adults aged 20–55 years and examined for *S. haematobium* infections as described elsewhere in detail [12, 14, 21]. In brief, the head teacher of each study school was informed about the aim and purpose of the study and about the results of the previous study year in an annual meeting convened at the beginning of the school term. A few weeks later, each school was visited by the study team to register eligible children from grades 3 and 4, to randomly select ~ 100 participants, to invite them to participate in the study, to obtain their parents’ written informed consent, and finally to collect their fresh urine samples for laboratory examination.

Similarly, the sheha (community head) of each study community (shehia) was informed about the study and subsequently the community was visited by the study team. Households were selected by a simple randomization procedure and a present adult household member was invited to participate in the study [14]. Once written informed consent was obtained, the participants were interviewed with a pretested questionnaire concerning demographic characteristics and MDA compliance, and invited to submit their own fresh urine sample.

All urine samples were collected between 10:00 AM and 2:00 PM and examined in the laboratory of the Zanzibar Neglected Diseases Programme in Unguja or at PHL-IdC in Pemba, respectively, during the later afternoon of the day of collection.

### Laboratory procedures

Upon arrival in the laboratory, the urine samples were sorted by increasing participant ID and examined for microhaematuria using reagent strips (Hemastix; *Siemens* Healthcare Diagnostics Ltd., Camberley, United Kingdom). Microhaematuria was coded semi-quantitatively in line with the colour reaction of the reagent strips and the manufacturer’s instructions as negative; trace; +; ++; and +++. Subsequently, urine samples with sufficient volume were vigorously shaken, and 10 ml were filtered through a filterholder containing a polycarbonate filter with a pore size of 20 μm (Sterlitech, Kent, WA, United States of America) using a standard 10 ml plastic syringe. The filter was then carefully transferred to a microscope slide, which was labeled with the participant ID, covered with hydrophilic cellophane soaked in glycerol, stained with Lugol’s iodine and examined under the microscope for the presence and number of *S. haematobium* eggs by trained laboratory technicians [7]. Finally, on the day of collection, 10 ml of 10% of all urine samples were filled into Falcon tubes and stored in a − 20 °C freezer in Pemba for future use. Several hundreds of the frozen urine samples were transferred to Switzerland on dry ice for further examination with the qPCR approach described below.

### qPCR system

#### Sample selection

A total of 1864 urine samples collected in Zanzibar between 2011 and 2017, were shipped on dry ice from Zanzibar to Basel in 2016 and 2017. Among them, 800 samples were selected by the study statistician for qPCR based examination at Biolytix AG in 2018, following these criteria: first, all samples from 2016 and 2017 were selected because there were only few samples available from those years; second, we selected all *S. haematobium* egg-positive samples and matched an egg-negative sample based on year, location, and survey (school/community) of collection. Finally, we randomly selected 585 samples to reach a total of 800 samples for examination with qPCR.

#### Sample preparation for the ring trial

For proficiency testing of the robustness of DNA isolation from urine and the *Dra1* based qPCR, eight laboratories based in Europe and Africa were invited to test urine samples for *S. haematobium Dra1* DNA in 2017. Two laboratories were based in sub-Saharan Africa (one in Tanzania and one in South Africa), and six in Europe (one in Germany, one in The Netherlands, one in Norway, two in Switzerland, and one in the United Kingdom). Among them, two were hospital laboratories, three were industry laboratories, and three were academic research laboratories. The aim of the ring trial was to explore how the DNA isolation and qPCR performed in different laboratories with different technicians and thermocyclers. Urine samples, the DNA Isolation Kit material, and the PCR Kit, respectively, were pooled and aliquoted at Biolytix AG before sending them to the ring trial participants to provide the same conditions for all participants. The urine samples (U1–U8) were sent in duplicates: one egg-negative (U1 ≙ U3) and three *S. haematobium* egg-positive samples (U7 ≙ U6, U2 ≙ U4, U5 ≙ U8). Hence, in total, eight samples were included in the test material. The participant laboratories were blinded to the infection status. In addition to the samples, one negative control (water as non-template control) and one positive control (sample containing *Dra1* DNA), the Kit material and a detailed protocol for DNA isolation and examination were provided to the participants. Once the test samples and material were received, ring trial participants isolated DNA from the urine samples using the provided *Quick*-DNA Urine Kit (D3061) and protocol as described below. The isolated DNA was analyzed by the multiplex qPCR system described below. Each sample was run on a thermocycler in 1:10 dilution and undiluted. Results were assessed qualitatively for the presence or absence of DNA of the target organism, *S. haematobium*, and the reference gene 18S rRNA. All participants submitted their qPCR results to Biolytix AG within three to 16 weeks. The results included the cycle threshold (Ct)-values of the qPCR analysis and the type of thermocycler used.

#### DNA isolation and purification for qPCR

DNA was extracted from the urine samples using the commercially available *Quick*-DNA Urine Kit (D3061) from Zymo Research according to the manufacturer’s protocol for cellular and cell-free DNA precipitation, but using an elongated protein digestion time and using the Zymo-Spin I-96 Plate (C2004) instead of the Zymo-Spin IC-S Column.

First, for DNA precipitation, 4 ml of each urine sample were mixed with 280 μl of urine conditioning buffer and vortexed. Subsequently, 10 μl of clearing beads were added and vortexed well. Finally, samples were spun for 15 min at 3000×*g* in a Hermle Z366 Centrifuge (Machinenfabrik Berthold Hermle AG, Gosheim, Germany).

Second, for protein digestion, the supernatant was carefully discarded at low speed (leaving around 100 to 200 μl liquid on top of the pellet). Subsequently, one volume of urine pellet digestion buffer was added to the mix. The pellet was then resuspended, and the mix suspended. Finally, 20 μl of Proteinase K were added to the suspension and incubated for 1 h at 55 °C in a shaking water bath. Third, for DNA purification, one volume of Genomic Lysis buffer was added to the digested mix (420 μl) and vortexed. Subsequently, the mix was transferred in a new 2 ml microcentrifuge tube, the sample was spun for 1 min at 3000×*g* in a Heraeus Pico 17 Centrifuge (Thermofisher Scientific, Waltham, United States of America), and the cleared supernatant was transferred into a new 2 ml microcentrifuge tube. Then, the samples were transferred into a Zymo-Spin I-96 plate (C2004, Zymo Research, Irvine, United States of America) on top of a collection box and spun for 2 min at 5796×*g* in a Sigma 4–15C plate centrifuge (Qiagen, Hilden, Germany). The Zymo-Spin I-96 plate was placed onto a new collection block, 200 μl of urine DNA prep buffer were added to each well with a multichannel pipette, and the plate was spun for 2 min at 5796×*g.* Subsequently, the Zymo-Spin I-96 plate was placed onto a new collection block, 700 μl of urine DNA wash buffer were added in each well with the multichannel pipette and the plate was spun for two minutes at 5796×*g*. This step was repeated once, using 200 μl of the same urine DNA wash buffer. Then, the plate was transferred on top of a 96-well elution tube rack and DNA was eluted with 100 μl of DNA elution buffer prewarmed at 60 °C. Finally, the plate was spun for 2 min at 5796×*g* and the elution plate containing extracted DNA was covered with an adhesive aluminum sticker cover and labelled with a unique barcode number.

#### Real-time PCR

The primers used for the qPCR for detection of the *Dra1* repeat unit in *S. haematobium* were selected based on the publication of Hamburger et al. 2001 [22]. To increase the sensitivity of the published PCR system from Hamburger et al. 2001 we used the probe published by Cnops et al. (2013) [23]. Moreover, the qPCR system was further optimized by adding an internal control detecting eukaryotic 18S rRNA in a multiplex qPCR. The sequences for *S. haematobium* detection were as follows: Sh_F; 5′-GATCTCACCTATCAGACGAAAC-3′, Sh_R 5′-TCACAACGATACGACCAAC-3′, and Sh_P; 5′-(FAM)-TGTTGGTGGAAGTGCCTGTTTCGCAA-BHQ1–3′. The sequences for the 18S rRNA detection were as follows: 18S_F; 5′-CGGCTACCACATCCAAGGAA-3′, 18S_R; 5′-CTATTGGAGCTGGAATTACCGC-3′, and 18S_P; 5′-(YY)-TGCTGGCACCAGACTTGCCCTCC-BHQ1–3′. The reaction was carried out in 20 μl containing 1× Takyon ROX Probe MasterMix UNG (Eurogentec, Seraing, Belgium), 160 nmol/L of Sh_F and Sh_R as well as 80 nmol/L of Sh_P, 40 nM of 18S-F and 18S_R as well as 20 nmol/L 18S_P, and 4 μl of target DNA. The participants of the ring trial used the thermocyclers available in their laboratory for the proficiency testing of the PCR system. The multiplex qPCR to examine the individual samples from Zanzibar for the second part of this study was carried out at Biolytix AG with an ABI 7900HT thermocycler (ThermoFisher Scientific, Waltham, United States of America) with a 384 well block module using the thermal profile including 2 min at 50 °C for the UNG activity, 10 min at 95 °C followed by 50 cycles of 15 s at 95 °C, and 60 s at 60 °C.

### Data management and analysis

The results of the macro- and microhaematuria grading and the number of *S. haematobium* eggs counted on the polycarbonate filter in 10 ml urine were recorded on paper sheets by the laboratory technicians and entered into a Microsoft Excel (Microsoft Corporation 2010) database by data entry clerks in Zanzibar. The results of the qPCR examination were directly obtained in electronic format in Switzerland. Data were analysed using STATA version 14.0 (StataCorp., College Station, TX, USA). Only data from urine samples with complete microhaematuria, urine filtration, and qPCR result were included in the analyses.

Urine samples with a reagent strip colour reaction indicating trace, +, ++ or +++ were defined as microhaematuria-positive. Samples, where the single urine filtration microscopy revealed at least one *S. haematobium* egg per 10 ml urine were considered as *S. haematobium* egg-positive. In line with WHO recommendations, *S. haematobium* infections were classified into light (1–49 eggs per 10 ml urine) and heavy (50+ eggs per 10 ml urine) intensity [1]. Additionally, to assess the performance of the qPCR at very low egg counts, we further stratified egg counts in the light infection intensity class into the following sub-classes: 1–5 eggs/10 ml, 6–10 eggs/10 ml, 11–49 eggs/10 ml. The DNA of each sample was diluted 1:2 and 1:20, respectively, and two Ct-value measurements per sample were taken for each dilution. The lowest Ct-value among the measurements was taken into account for further analysis. Ct-values of < 33 derived after 50 qPCR amplification cycles were considered as *Dra1* DNA-positive. The sensitivity of the qPCR was calculated as the proportion of *Dra1* DNA-positives that were correctly identified when compared with the results of the reference test. Reference tests, though imperfect since they lack sensitivity to detect very light intensity infection [7], were i) urine filtration microscopy, ii) reagent strip tests, and iii) combined results of urine filtration microscopy and reagent strip test. Sensitivity was also calculated stratified by the different egg count (sub-) classes to assess the performance of the qPCR at very light intensity infections. Correlation between Ct-values derived after 50 amplification cycles of the qPCR and the number of *S. haematobium* eggs per 10 ml urine were assessed with the Spearman’s rank correlation test.

## Results

### Inter-laboratory robustness of qPCR

All eight laboratories that were invited did participate in the ring trial. The eight laboratories used five different types of thermocyclers for the qPCR (ABI 7500, ABI 7900HT, Agilent Technologies Stratagene MX3005P, Bio-Rad CFX96 Real-Time System, Roche LightCycler Nano).

As indicated in Table 1, all eight laboratories correctly identified the six *S. haematobium* egg-positive urine samples as *Dra1* DNA-positive. Considering Ct-values of ≥33 as negative, the two egg-negative samples were correctly identified by seven among the eight laboratories. One laboratory obtained Ct-values of 30.0 and 31.9, respectively, for these negative samples when diluted 1:10 and Ct-values of 26.7 and 28.1 for the undiluted samples, which we considered as an incorrectly positive result.

**Table 1:** Cycle threshold (Ct)-values of the qPCR analysis performed by different laboratories to detect *S. haematobium Dra1* DNA in eight different urine sample pools, respectively

Code	(1:10 dilution *Dra1*)	Laboratory 1	Laboratory 2	Laboratory 3	Laboratory 4	Laboratory 5	Laboratory 6	Laboratory 7	Laboratory 8
U1	Urine, *Schistosoma* egg-negative	no-amp	no-amp	no-amp	no-amp	no-amp	0.0	37.7	30.0
U2	Urine, *Schistosoma* egg-positive	21.5	20.4	22.6	19.2	20.4	21.6	21.3	18.2
U3	Urine, *Schistosoma* egg-negative	47.5	no-amp	no-amp	no-amp	no-amp	0.0	36.9	31.9
U4	Urine, *Schistosoma* egg-positive	21.4	20.7	21.2	18.5	19.3	22.7	20.7	19.7
U5	Urine, *Schistosoma* egg-positive	20.4	22.2	22.3	22.9	26.0	19.5	22.1	24.5
U6	Urine, *Schistosoma* egg-positive	23.6	18.8	18.8	19.1	18.6	22.0	19.8	20.8
U7	Urine, *Schistosoma* egg-positive	18.6	18.6	21.1	16.3	17.3	19.4	20.6	20.2
U8	Urine, *Schistosoma* egg-positive	25.7	27.0	28.1	25.5	26.6	24.8	27.7	27.6
PC	Positive Control	22.6	24.6	23.2	21.9	23.3	20.13	27.4	23.7
NTC	No Template Control	no-amp	no-amp	38.3	no-amp	no-amp	no-amp	no-amp	no-amp
	Thermocycler	Abi7900	ABI 7500	Bio-rad CFX 96	Stratagene MX3005P	Bio-rad CFX 96	Bio-Rad CFX 96	Bio-Rad CFX 96	Roche LightCycler Nano

*no-amp* No DNA amplification up to cycle 50. Ct-values of ≥33 were considered as negative

### Characteristics of urine samples from Zanzibar

**Fig. 1:**
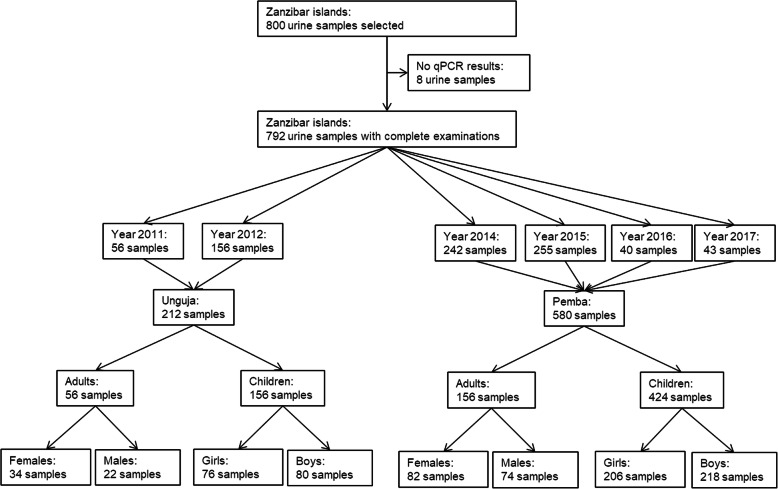
Characteristics of urine samples from children and adults from Pemba and Unguja islands, United Republic of Tanzania, that were analysed with qPCR

Urine samples from a total of 800 individuals from Zanzibar were selected for examination with qPCR in 2018 (Fig. 1). A total of 792 samples had complete examinations with urine filtration, reagent strips, and qPCR. Among the 212 samples with complete examinations collected in Unguja, 56 were from adults and 156 were from children. Among the 580 samples with complete examinations collected in Pemba, 156 were from adults and 424 were from children. *S. haematobium* eggs were identified by single urine filtration microscopy in 13.3%, microhaematuria by reagent strips in 13.8%, and *S. haematobium* DNA by qPCR in 26.8% of the urine samples.

### Sensitivity of qPCR with urine filtration microscopy and/or reagent strips as reference method

As indicated in Table 2 and Tab. S1, in total, the qPCR detected more than twice as many urine samples (212/792) as *Schistosoma*-positive than the urine filtration (105/792) and the reagent strips (109/792), respectively. Among the 212 *Dra1* DNA-positive samples, 44.3% (94/212) were egg-positive, 40.6% (86/212) were microhaematuria-positive, and 48.1% (102/212) were either egg-positive or microhaematuria-positive.

**Table 2:** Sensitivity and specificity of qPCR using urine filtration, reagent strips, or a combination of urine filtration and reagent strips as reference test for *S. haematobium* diagnosis in urine samples from Zanzibar

	Urine filtration	Negative	Positive	Total
**qPCR**	Negative	569	11	580
	Positive	118	94	212
	Total	687	105	792
	Sensitivity	89.5% (95% *CI*: 87.4–91.7%)		
	Specificity	82.8% (95% *CI*: 80.2–85.5%)		
	**Reagent strips**	Negative	Positive	Total
**qPCR**	Negative	557	23	580
	Positive	126	86	212
	Total	683	109	792
	Sensitivity	78.9% (95% *CI*: 76.1–81.7%)		
	Specificity	81.6% (95% *CI*: 78.9–84.3%)		
	**Urine filtration plus reagent strips**	Negative	Positive	Total
**qPCR**	Negative	553	27	580
	Positive	110	102	212
	Total	663	129	792
	Sensitivity	79.1% (95% *CI*: 76.2–81.9%)		
	Specificity	83.4% (95% *CI*: 80.8–86.0%)		

*95% CI* 95% confidence interval

**Fig. 2:**
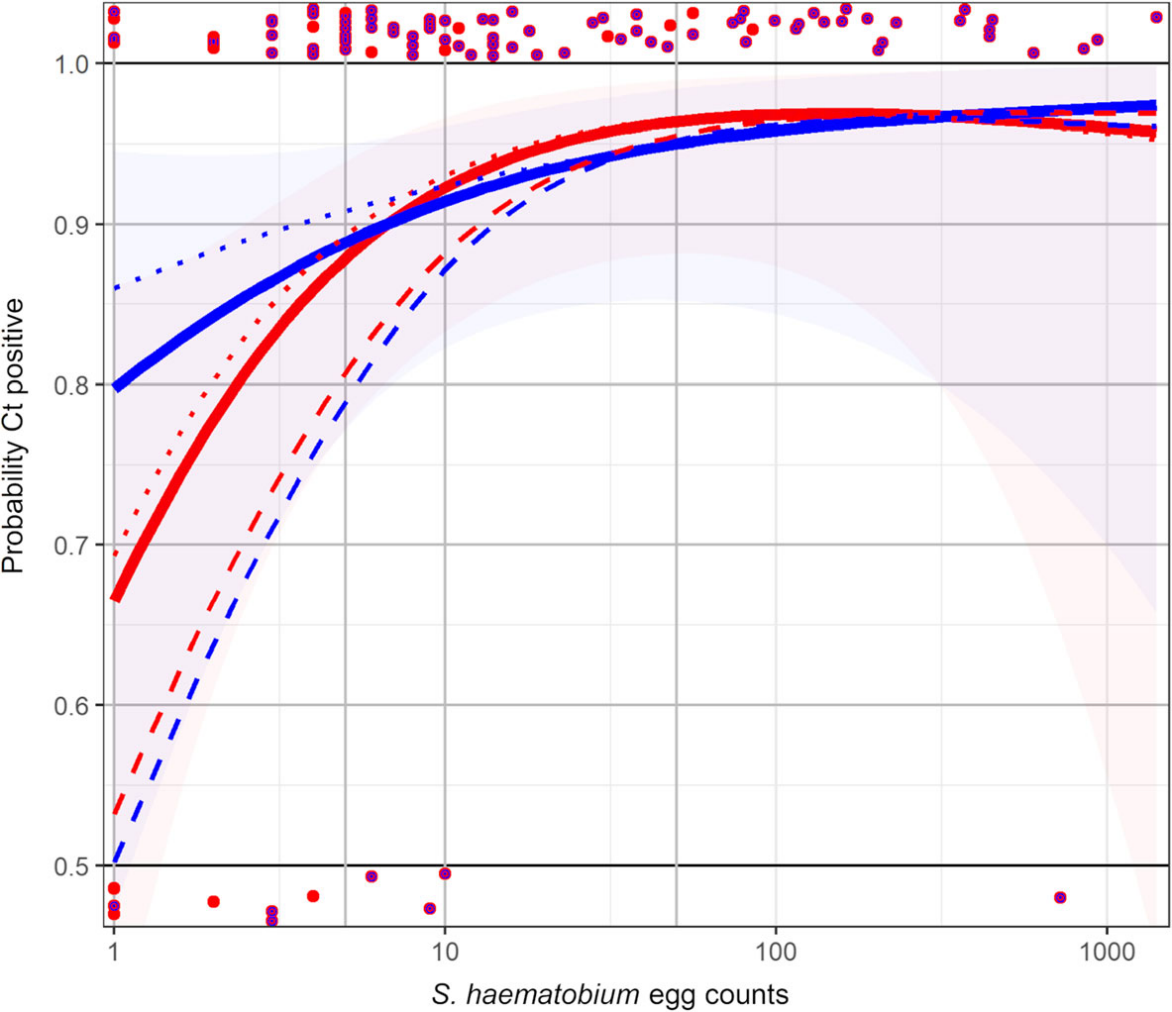
Probability of a positive qPCR Ct-value in relation to increasing *Schistosoma haematobium* egg counts from urine filtration microscopy. Red dots: *S. haematobium* egg counts in qPCR-positive (top) or qPCR-negative negative (bottom) samples, when Ct-values of < 33 are considered as positive; red and blue dots: *S. haematobium* egg counts and microhaematuria in qPCR-positive or qPCR-negative negative samples, when Ct-values of < 33 are considered as positive; red line: probability curve for Ct-values < 33 to become positive if urine filtration is the reference test; blue line: probability curve for Ct-values < 33 to become positive if urine filtration plus reagent strip tests are the reference test; dotted red line: probability curve for Ct-values < 34 to become positive if urine filtration is the reference test; dotted blue line: probability curve for Ct-values < 34 to become positive if urine filtration plus reagent strip tests are the reference test; dashed red line: probability curve for Ct-values < 32 to become positive if urine filtration is the reference test; dashed blue line: probability curve for Ct-values < 32 to become positive if urine filtration plus reagent strip tests are the reference test

When the urine filtration was used as reference method, the qPCR identified *Dra1* DNA in 89.5% (94/105) of *S. haematobium* egg-positive samples. As shown in Fig. 2 and Tab. S1, the probability of a positive *Dra1* DNA result increased with increasing egg counts. The sensitivity of the qPCR was 80.6% (29/36) for counts between 1 and 5 eggs, 83.3% (15/18) for counts between 6 and 10 eggs, 100% (23/23) for counts between 11 and 49 eggs and 96.4% (27/28) for counts of 50+ eggs. The one egg-positive sample which was not identified as positive in the last group had a count of 721 eggs. With regard to egg-count infection intensity categorizations defined by the WHO, the sensitivity of qPCR to detect light and heavy infection intensities was 87.0% (67/77) and 96.4% (27/28), respectively.

When reagent strips were used as reference method, the qPCR identified *Dra1* DNA in 78.9% (86/109) of microhaematuria-positive samples. The sensitivity for *Dra1* DNA detection was higher when the microhaematuria grading was ++ (81.5%, 22/27) and +++ (86.1%, 31/36), compared with trace (75.0%, 21/28) and + (66.7%, 12/18).

Compared with the combined results of urine filtration and reagent strips as reference method, the qPCR had a sensitivity of 79.1% and a specificity of 83.4%.

### Characteristics of urine samples with false-negative qPCR results

Tab. S2 shows the characteristics of the 27 urine samples that had *S. haematobium* egg counts in the urine filtration microscopy or microhaematuria-positive reagent strip results, but were negative for *Dra1* DNA in the qPCR. Among them, 11 samples were identified as *S. haematobium* egg-positive by urine filtration but negative for *Dra1* DNA by qPCR. Among the 11 samples, seven had egg counts ranging between 1 and 4. When the microscope slides were read a second time for five among those seven slides, four had zero egg counts, but one among those four was microhaematuria-positive. Among these 11 samples, four had egg counts ranging between six and 721 eggs, mostly confirmed by a second reading of the microscope slides and all were microhaematuria-positive.

Among the 16 samples that were microhaematuria-positive but egg-negative and *Dra1* DNA-negative, 13 were from females (three girls and 10 adult females).

### qPCR Ct-values as quantitative measure for infection intensity

There was a significant negative correlation between Ct-values measured by qPCR and *S. haematobium* eggs counted under the microscope (spearman’s rho = -0.49, *P* < 0.001).

**Fig. 3:**
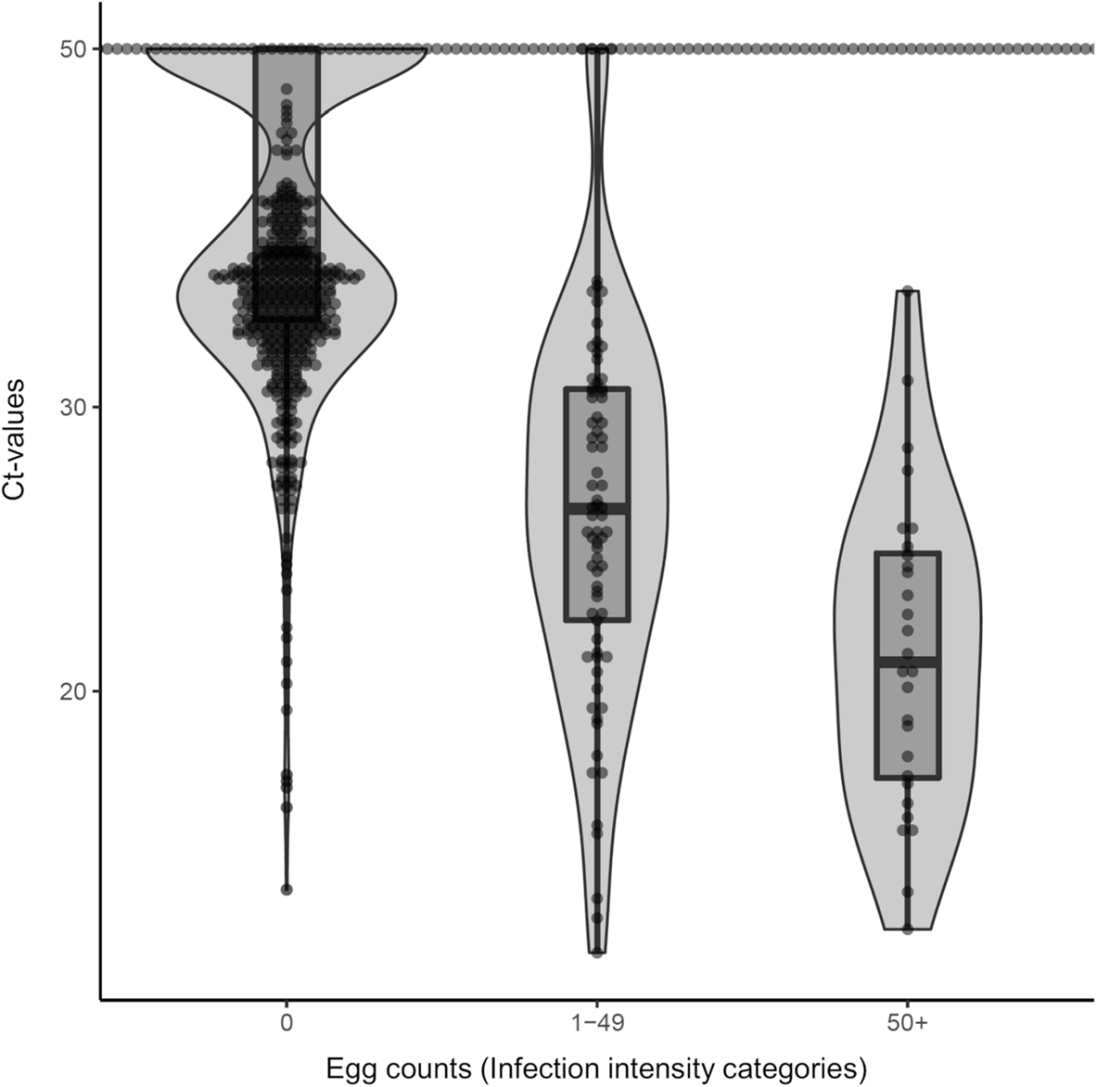
qPCR Ct-values of samples stratified by infection intensity categories based on their *S. haematobium* egg counts (0 eggs = negative, 1–49 eggs = light infection; 50+ eggs = heavy infection). Boxes represent 25, 50 and 75% quintiles. Whiskers present minimum and maximum without outliers

The median and first and third quartiles of Ct-values of samples when stratified by WHO defined infection intensity categories based on *S. haematobium* egg counts is illustrated in Fig. 3. In the egg-negative class, 75% of the Ct-values were ≥ 33 with a median of 37. In the light intensity egg-count class, 75% of the Ct-values were < 31 with a median of 26. In the heavy intensity class, 75% of the Ct-values were < 24 with a median of 21. While the medians followed a decreasing trend, some overlap was observed.

### *S. haematobium*-positive rate in Zanzibar by qPCR

**Fig. 4:**
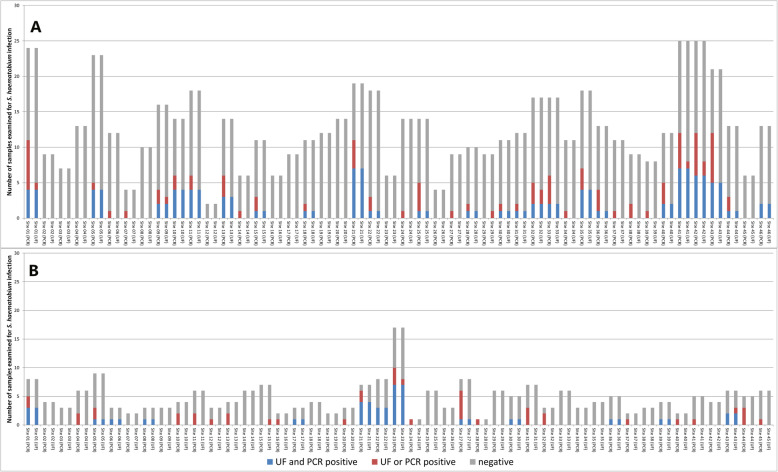
Number of samples examined and identified as *S. haematobium*-positive with urine filtration (UF) and/or qPCR in 46 study sites in Pemba (**a**) and 45 study sites in Unguja (**b**) islands, United Republic of Tanzania

The selection procedure for the 792 samples examined with qPCR was not designed to reveal a *S. haematobium* prevalence for Zanzibar in general or any of the study locations in particular. Figure 4a and b show the number of samples examined and the number of samples that were negative, egg-positive and/or *Dra1* DNA-positive for each study site in Pemba and Unguja, respectively. A total of 223 egg-positive and/or *Dra1* DNA-positive urine samples were identified in 34/46 locations in Pemba and in 30/45 locations in Unguja.

Among those 223 samples, 94 samples were recognized as *S. haematobium*-positive by both urine filtration and qPCR, 118 samples were diagnosed as positive by qPCR only, and 11 samples were identified by urine filtration only (Table 2). Among the 118 *Dra1* DNA-positive only samples, 69 were samples from 23 sites in Pemba and 14 were samples from 5 sites in Unguja, where also additional, both egg-positive and *Dra1* DNA-positive samples came from. The remaining 35 samples were from 16 locations in Unguja (*n* = 25) and nine locations in Pemba (*n* = 10), where no egg-positive samples were identified by urine filtration. Among the 11 egg-positive samples that were only identified by urine filtration but not qPCR, nine were from seven sites, where also additional egg-positive or *Dra1* DNA-positive samples were identified and two samples were from one site in Pemba and Unguja each, where no other positives were found.

## Discussion

The WHO envisions a world free of schistosomiasis [1]. Mass drug administration and complementary interventions to control and eliminate schistosomiasis in endemic areas have been scaled up over the past decades [5, 13, 24–26]. Moving towards elimination, sustained efforts will result in decreased *Schistosoma* prevalence and infection intensities [12, 27–29], and diagnosis of *Schistosoma* infections will become harder with standard classical parasitological methods [7]. Monitoring the impact of interventions in elimination settings, surveillance to avoid recrudescence of infection, and verification of interruption of transmission require more sensitive, robust, and high-throughput approaches for *S. haematobium* diagnosis. Here, we assessed the performance of a standardized qPCR approach for *S. haematobium* diagnosis in a multi-laboratory ring trial and by examination of 792 urine samples collected in Zanzibar, one of the first settings in sub-Saharan Africa targeted for schistosomiasis elimination.

The ring-trial involving a total of eight research, hospital or biotech laboratories from Europe and Africa showed that all *S. haematobium* egg-positive samples were correctly identified by qPCR in all participating laboratories. Importantly, the results were comparable by running the qPCR on five different brands and types of thermocyclers. One laboratory obtained false-positive results in the egg-negative urine samples. Hence, while the standardized qPCR approach can be considered as accurately detecting *S. haematobium*-positive samples, the occurrence of false-positive samples could not be ruled out.

Applying the qPCR for *S. haematobium* diagnosis in urine samples from Zanzibar, *Dra1* DNA detection by qPCR clearly outperformed egg detection by urine filtration microscopy and microhaematuria assessment by reagent strips. The qPCR identified twice as many samples as *S. haematobium*-positive compared with the classical diagnostic methods. Most of the samples (108/118) that were *Dra1* DNA-positive and *S. haematobium* egg-negative had Ct-values < 33 and > 22 and might hence be regarded as light intensity infections. While it is known that particularly very light intensity infections are often missed by reagent strips or urine filtration microscopy [7], it might also be that qPCR detects excreted parasite DNA in the absence of egg production. In that case, old egg granulomas might exist, which still release DNA. Or, worms could be present but not produce eggs at all or only intermittently. Circadian and daily variation in egg excretion have been reported for *S. haematobium* [30–32]. Hence, infection intensity classification based on egg counts from a single urine filtration from one day can be considered a meaningful measure at population level but not at individual level. Ct-levels from *Schistosoma* DNA have been reported to be more stable over multiple days than egg counts [32] and also our ring trial confirmed little variation between the different laboratories and technicians. Ct-values might therefore serve as a better measure of infection intensity than egg counts and might in future be used for evaluation of control and elimination programs as well as for drug efficacy assessment.

However, not all egg-positive and/or microhaematuria-positive urine samples were identified as *Dra1* DNA-positive. Most of these false-negative samples (7/11) had extremely low egg counts (below five eggs per 10 ml urine) and some of them were not confirmed by quality control readings of the same slide. Hence, as suggested for false-negative results in other studies, the amount of DNA in these very lightly infected samples might have been below the detectable limit [32, 33]. However, four samples had counts between six and 721 eggs and it is not clear, why no DNA amplification occurred. There was no indication that storage time since sample collection, location of the participant’s residence, sex or the microhaematuria level influenced the qPCR outcome in our study here, but labelling errors in 3% of samples have been suggested as potential reason for discrepant diagnostic results in another study from Zanzibar working with urine samples from the same surveys [7]. Most of the microhaematuria-positive but egg-negative and *Dra1* DNA-negative urine samples were from females, and the explanation that the microhaematuria was caused by menstruation blood or other infections of the urogenital tract is self-evident. Clearly, not all microhaematuria is caused by schistosomiasis [34].

Using urine filtration microscopy as reference method, the qPCR had a sensitivity of 89.5%. This value is greatly in line with previous assessments of qPCR systems for *S. haematobium* [32, 33, 35, 36]. Also the sensitivity of our qPCR approach for light (87.0%) and heavy (96.4%) intensity infections matches very well with the sensitivity revealed by Obeng et al. [33]. Going beyond the WHO classifications, we found that the sensitivity was 80.6 and 83.3% at egg counts of 1–5 and 6–10 eggs per 10 ml urine, respectively, and that the sensitivity reached (almost) 100% at counts > 10 eggs per 10 ml.

The examination of urine samples from 91 study sites in Zanzibar showed that the qPCR mostly identified additional *S. haematobium*-positive individuals in locations, where also the urine filtration microscopy identified cases. Hence, in these places the *S. haematobium* prevalence is likely underestimated when urine filtration is used as diagnostic approach, and might be twice as high as assumed. Moreover, the qPCR identified some few cases in sites where all urine filtration examinations were negative. Both observations are in line with an earlier study using the highly sensitive up-converting phosphor lateral flow circulating anodic antigen (UCP-LF CAA) assay in Pemba, which showed that there are far more individuals infected with *S. haematobium* than estimated by the urine filtration [11]. The results have important ramifications for future elimination efforts on the islands. Clearly, the true prevalence is considerably higher than the apparent prevalence and schistosomiasis elimination interventions need to be sustained to avoid a rebound of infections and to advance the gains that have been made in Zanzibar to date. Mass drug administration to further reduce the *S. haematobium* prevalence might be justified for an extended period, at least in areas where urine filtration shows a reasonably high prevalence (and the true prevalence might again be higher). If, as suggested elsewhere [12, 13], the Zanzibar schistosomiasis elimination programme, guided by donor decisions and research outcomes based on urine filtration, considers changing the tactics from MDA to more tailored intervention approaches such as surveillance-response including test and treat approaches, a sensitive diagnosis should be warranted. Ideally, the new diagnostic test should be applicable at the point of care, i.e. not only in a well-equipped central laboratory, but also at the peripheral level in health facilities, hospitals, and within control and elimination programs in basic laboratories, schools and households. It should also be rapid and high-throughput so that infected individuals can be tested at the spot and treated immediately if positive. DNA-based tests with point-of-care application include the loop-mediated isothermal amplification (LAMP) [37, 38] and the recently developed recombinase polymerase amplification (RPA) [39] assays. The qPCR approach used in this study is not a rapid diagnostic test that can be used at peripheral level in facilities without additional equipment and electricity. It requires a well-equipped laboratory, experienced and organized technicians, and a considerable amount of time, particularly for DNA extraction, if many samples are to be tested. If not for routine surveillance in endemic countries, qPCR, in an improved format and with further increased sensitivity and specificity so that also ultra-light intensity infections are reliably detected, may yet be the approach of choice for verification and certification of elimination, once successful schistosomiasis elimination programmes need the proof of interruption of transmission. Moreover, a standardized and highly sensitive and specific qPCR approach will be highly useful to measure the impact of control and elimination interventions in low-prevalence settings in endemic countries where it will be able to more accurately assess the prevalence and incidence levels than classical parasitological methods. Also, qPCR might have a role to play in the detection of female genital schistosomiasis when urine egg counts are low. Finally, the application of qPCR can be recommended for a clinical environment in Europe and the United States, where individual diagnosis is important and where migrants, travellers and tourists may present with very light intensity or prepatent infections that may be missed by classical parasitological diagnosis.

## Conclusions

The qPCR approach presented here is a standardized and sensitive approach for *S. haematobium* diagnosis that can be used for impact assessment of schistosomiasis elimination programmes, individual diagnosis of *S. haematobium* infection, and in improved format also for verification and certification of elimination.

## Supplementary information

**Additional file 1: Table S1.** showing the multivariate frequency distribution of *S. haematobium* infection measured by urine filtration (egg counts), reagent strips (microhaematuria), and qPCR (*Dra1* DNA).

**Additional file 2: Table S2.** showing the characteristics of 27 urine samples that had *S. haematobium* egg counts in the urine filtration (UF) microscopy or microhaematuria-positive reagent strip results, but were negative for *Dra1* DNA in the qPCR.

## Data Availability

The data supporting the conclusions of this article are included within the article and its additional files. The datasets generated and/or analysed during the current study are available from the corresponding author upon reasonable request.

## Abbreviations

MDA: Mass drug administration
MoH: Ministry of Health
PHL-IdC: Public Health Laboratory-Ivo de Carneri
qPCR: Real time-polymerase chain reaction
WHO: World Health Organization
ZEST: Zanzibar Elimination of Schistosomiasis Transmission
